# Randomized, Double‐Blind, Controlled Study to Evaluate Safety and Pharmacokinetics of Single Ascending Doses of ASP5354, an Investigational Imaging Product, in Healthy Adult Volunteers

**DOI:** 10.1002/cpdd.1013

**Published:** 2021-08-23

**Authors:** Tosei Murase, Masaomi Takizawa, Lawrence Galitz, Stephen Flach, Valene Murray, Brandon Gufford, Akira Suwa

**Affiliations:** ^1^ Astellas Pharma Inc Tokyo Japan; ^2^ Labcorp Drug Development Inc. Daytona Florida USA; ^3^ Labcorp Drub Development Inc Madison Wisconsin USA; ^4^ Labcorp Drug Development Inc Leeds UK; ^5^ Rx+ Business Accelerator Astellas Pharma Inc Ibaraki Japan

**Keywords:** ASP5354, iatrogenic ureteral injury, indocyanine green, near‐infrared fluorescence, ureter visualization

## Abstract

Intraoperative ureter identification helps reduce the risk of ureteral injury. Currently, no suitable agents for real‐time ureter visualization are approved. ASP5354 (TK‐1) is a novel indocyanine green derivative. In this first‐in‐human phase 1, double‐blind, sequential ascending‐dose study, urethral catheters were placed in 6 healthy volunteers who were randomized to single‐dose, intravenous ASP5354 0.1 mg (n = 4) or placebo (n = 2). Sequential dose escalations to 0.5‐, 2‐, 8‐, and 24‐mg ASP5354 in new cohorts were contingent upon Dose‐Escalation Committee approval after review of pharmacokinetic (PK) and safety data. Blood and urine samples were collected over 24 hours following dose administration. Objectives were to assess the safety/tolerability and PK of ASP5354. Treatment‐emergent adverse events (TEAEs) were reported in 3 (15%) and 2 (20%) participants in the ASP5354 and placebo groups, respectively. In the former, there were 6 TEAEs (5/6 grade 1‐2). One ASP5354 participant experienced grade 3 pyelonephritis, attributed to the catheter. No TEAEs were related to ASP5354. Mean plasma terminal elimination half‐life ranged from 2.1 to 3.6 hours, with near complete urinary excretion of unchanged ASP5354 within 24 hours after administration. Linear and dose‐proportional PK were observed. These results support further evaluation of ASP5354 at doses up to 24 mg for intraoperative near‐infrared fluorescence ureter visualization.

Although ureteral injury is an infrequent occurrence, 75% of all cases occur during abdominal or pelvic surgery,^1^ largely as a consequence of the close proximity of the ureter to anatomic structures encountered during the procedure.^2^ Most iatrogenic ureteral injuries (IUIs) stem from gynecological procedures,^3^ where rates of 0.1% to 1.5% have been reported for nononcologic surgeries.^4^ Colorectal surgery is the second most common source of IUIs. An analysis of >2 million colorectal surgeries performed in the United States over 10 years identified a 0.28% rate of IUIs, representing 6027 injuries.^5^ If not detected and treated promptly, IUIs can have serious sequelae, increasing morbidities such as ureteral strictures and reduced long‐term renal function,^6^ and contributing to longer hospital stays, increased hospital costs, and increased mortality.^5^ In addition, IUIs are not without medicolegal and financial implications for the surgeon.^7^ For IUIs, the single greatest prognostic factor is time to diagnosis; superior outcomes are associated with intraoperative diagnosis and repair.^4^, ^8^

Detection of IUI, however, can be difficult, and only about one‐third of IUIs are diagnosed intraoperatively.^1^, ^8^, ^9^, ^10^ Prevention of IUI—by far the most desirable course—is often stymied by the challenges involved in ureter identification, particularly during laparoscopic procedures.^11^, ^12^, ^13^ Prophylactic ureteral stenting, which aids in ureter visualization, is sometimes employed in complex procedures^14^ but remains controversial and has the potential itself to induce IUIs.^5^ Although considered acceptable in high‐risk procedures, current guidelines do not call for its routine use.^8^, ^15^ Preoperative imaging techniques such as intravenous (IV) urography and computed tomography can be used but do not provide real‐time visualization and may not prevent IUIs.^8^ Clearly, better noninvasive methods of ureter identification are needed, and 1 survey even found that the majority of surgeons (54.5%) would consider implementing such a technique in their regular daily practice.^14^

Near‐infrared fluorescence (NIRF) imaging is a promising technique for real‐time visualization of anatomic structures.^2^, ^12^, ^16^, ^17^, ^18^, ^19^ Preoperative injection of a renally excreted NIRF contrast agent that can be detected by intraoperative imaging systems allows for real‐time ureter visualization and avoidance without the use of radionuclides. Near‐infrared (NIR) light can penetrate through 5 mm of tissue,^18^ provide a strong visual signal due to low tissue autofluorescence and weak absorption in the NIR range,^18^ and does not change the visual appearance of the surgical field, thereby providing “an enhanced reality beyond standard white light visual inspection and palpitation.”^12^ Key to these desirable properties, however, is the contrast agent itself. The first‐in‐human study of this technique for ureter identification used the dye methylene blue,^19^ but this agent did not provide sufficient optical properties to offer any visualization advantage with NIR over white light.^16^ Improvements in NIFR contrast agents for ureter visualization has consequently been an area of active research.^12^

ASP5354 (TK‐1) is a novel indocyanine green derivative containing β‐cyclodextrin moieties.^20^ Its molecular size and hydrophilic nature allow for its excretion into urine, imparting a visibly green coloration and, with much greater sensitivity, enabling ureteral‐specific imaging and visualization using existing NIR indocyanine green detection devices.^21^ Preclinical results showed that IV ASP5354 at 0.01 mg/kg allowed visualization of ureters for up to 3 hours after administration.^22^ Briefly, the proportions of animals whose ureters were visible up to 3 hours after administration of ASP5354 chloride were 33% at 0.001 mg/kg and 100% at 0.01 mg/kg, respectively. In addition, ASP5354 was well tolerated, having no toxicity‐related changes when dosed once daily for 4 weeks of up to 300 mg/kg in cynomolgus monkeys.^22^

The present phase 1 study evaluated the safety and tolerability, as well as the pharmacokinetics (PK) of a single dose of ASP5354 administered to healthy volunteers.

## Methods

### Study Design

NCT03698305 was a randomized, double‐blind, placebo‐controlled, sequential ascending IV bolus dose group study conducted at a single center (Labcorp Drug Development [formerly Covance] Clinical Research Unit, Inc.) in the United States. The objectives were to assess the safety and tolerability of ASP5354 administered intravenously as a single dose to healthy participants and to assess the single‐dose PK profile of ASP5354 in plasma and urine.

**Figure 1:**
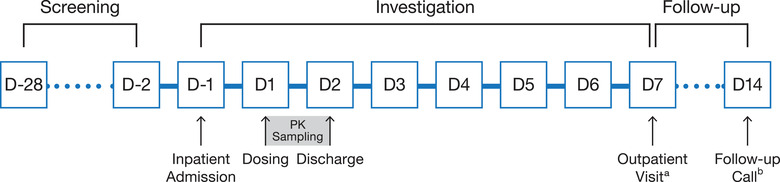
Study schema. ^a^Outpatient visit took place on day 7 or at early discontinuation from study. ^b^Follow‐up phone call took place on day 14 ± 3 days. PK, pharmacokinetic.

The study population, 30 participants in all, was composed of 5 cohorts consisting of 6 healthy volunteers (3 women and 3 men) per cohort. Participants were randomly assigned 2:1 (n = 4 and n = 2 in each cohort) to receive a single IV bolus dose of ASP5354 or placebo. The study consisted of a screening period, an investigational period, and a follow‐up period (Figure 1). Following successful screening, participants were admitted to the clinic on study day –1. On day 1, participants had an indwelling urethral catheter placed 1 to 2 hours before dosing, which remained until ≥8 hours after dosing. Under fasting conditions, participants in cohort 1 randomly assigned to ASP5354 received 0.1 mg of ASP5354 by IV bolus; subsequent cohorts received 0.5, 2, 8, and 24‐mg ASP5354 boluses, sequentially. The estimated clinical efficacious dose for ASP5354 is 0.5 mg per subject. In the imaging study of Göttingen minipigs, the ureter under fluorescent imaging was visually identifiable at >1 μg/mL of urinary concentration at 3 hours after IV administration of ASP5354.^22^ The efficacious dose in humans, therefore, was calculated as the dose that gives urine concentration above 1 μg/mL at 3 hours after IV administration of ASP5354. To evaluate the dose dependency of ASP5354 PK in the first‐in‐human study, the starting dose of 0.1 mg, which is 5‐fold lower than the estimated clinical efficacious dose (0.5 mg), was selected.

After at least 5 of 6 participants in a cohort had completed the study procedure, a Dose‐Escalation Committee reviewed all PK and safety data, and decided whether to proceed with dose escalation, stop dose escalation, repeat a dose level, or investigate a lower dose level intermediate between the current and prior doses. ASP5354 solution for injection was supplied as a 4 mg/mL aqueous solution for IV injection. ASP5354 solution was provided in 10‐mL amber glass vials. The rationale for dose levels can be found in the Supplemental Information.

Blood and urine samples were collected over a 24‐hour period. Participants were discharged on day 2 and returned to the clinic for a follow‐up visit on day 7, and the study was completed with a follow‐up phone call on day 14. To prevent accidental unblinding should the urine be discolored, catheter and tubing collection bags were covered, and participants were prevented from observing these or the collection vials when changed. Collection and processing of urine samples was conducted by a separate, additional unblinded staff that did not participate in the assessments.

Informed consent was obtained from each participant and the protocol was approved by the Institutional Review Board (Advarra Institutional Review Board, Columbia, Maryland). The study was further conducted in accordance with the principles of the Declaration of Helsinki, Good Clinical Practice, and International Council for Harmonisation of Technical Requirements for Pharmaceuticals for Human Use guidelines. Amendments made to the study protocol, discussed below (see Participants and Assessments sections), did not affect outcomes in this study.

### Participants

Eligible participants were 18 to 55 years of age, with a body mass index (BMI) of 18.5 to 32.0 kg/m^2^, inclusive, and weighing >40 kg (women) or >50 kg (men). Female participants were excluded if pregnant and were required to abstain from breastfeeding throughout the treatment period and for at least 30 days after final drug administration. All participants were required to follow contraceptive guidelines. All participants had normal liver function (alanine aminotransferase, aspartate aminotransferase, alkaline phosphatase, gamma‐glutamyl transferase, and total bilirubin less than the upper limit of normal) and normal renal function (blood urea nitrogen and creatinine less than or equal to the upper limit of normal) at day –1, the latter added as an amendment to the protocol on October 31, 2018. A history or evidence of clinically significant disease or malignancy was not permitted. All participants provided written informed consent.

### Assessments

Safety and tolerability were assessed at each dose level through monitoring of adverse events (AEs) associated with ASP5354, clinical laboratory tests, vital signs, electrocardiograms (ECGs), and physical examinations. End points for safety and tolerability were the nature, frequency, and severity of AEs and clinical laboratory tests, vital signs, routine 12‐lead ECGs, and physical examinations. If subjects developed a hypersensitivity reaction, an additional blood sample for determination of histamine concentration was taken as soon as possible after the onset of the hypersensitivity reaction.

AEs were graded according to the National Cancer Institute Common Terminology Criteria for Adverse Events, version 5.0, as specified in the October 31, 2018, protocol amendment. AEs were categorized by organ class and preferred term using the Medical Dictionary for Regulatory Activities, version 21.1. Green coloration of the urine was not considered an AE, as this was an expected and known reversible effect lacking any untoward clinical symptoms.

Time points for blood and urine PK sample collection are shown (Table 1). Urine interval sampling was between –1 hour and time of dosing, and between each subsequent consecutive urine sample collection time point. End points for PK parameters of ASP5354 in plasma were back‐extrapolated plasma concentration at time 0; maximum observed plasma concentration; area under the plasma concentration‐time curve (AUC) from time 0 to 24 hours after dosing, from time zero to time of last quantifiable concentration, AUC from time 0 to infinity (AUC_inf_), and AUC from time of last quantifiable concentration to infinity as percentage of total area under the concentration‐time curve; total body clearance of drug from plasma, time of maximum observed concentration; apparent terminal elimination half‐life; and volume of distribution during the terminal phase. End points for PK parameters of ASP5354 measured in urine were amount of unchanged drug excreted into the urine (Ae), percentage of dose excreted into the urine (Ae%), cumulative Ae, percentage of cumulative Ae, Ae from time 0 to the time of last quantifiable concentration (Ae_last_), percentage of Ae from time 0 to the time of last quantifiable concentration, renal clearance, and mean ASP5354 urine concentration at each time point. Details regarding methodology for the bioanalysis of ASP5354 can be found in the Supplemental Information.

**Table 1:** Postdose Time Points for PK Sample Collection (h)

Blood	Urine Point Samples	Urine Interval Collection
0.083	0.5	–1 to 0
0.25	1.0	0 to 0.5
0.5	2.0	0.5 to 1.0
0.75	3.0	1.0 to 1.5
1.0	4.0	1.5 to 2.0
1.5	6.0	2.0 to 2.5
2.0	8.0	2.5 to 3.0
3.0	24.0	3.0 to 3.5
4.0	…	3.5 to 4.0
6.0	…	4.0 to 6.0
8.0	…	6.0 to 8.0
24.0	…	8.0 to 12.0
…	…	12.0 to 24.0

PK, pharmacokinetic.

Start and stop interval times of green urine coloration were recorded for the 8 and 24‐mg ASP5354 cohorts per the April 17, 2019, protocol amendment. Detection of coloration in the urine was performed by unblinded nursing staff using visual inspection only. Coloration records were maintained in a secure location until after database lock to avoid potential unblinding of blinded staff.

### Statistical Methods

The safety population comprised all participants who received a single dose of the study drug. The PK population comprised safety population participants for which data were available for derivation of ≥1 PK parameter. PK parameters were calculated by noncompartmental analysis using Phoenix WinNonlin version 8.1 (Certara, Princeton, New Jersey) and were summarized by treatment group. Descriptive statistics are presented for plasma concentrations, amount and cumulative amount of ASP5354 excreted in urine, and ASP5354 concentrations for point urine collection by treatment group and scheduled sample time. Criteria for handling concentrations below the limit of quantification are detailed in the Supplemental Information.

## Results

### Participants

There were 30 participants, of whom 20 (4 per cohort) received ASP5354 and 10 (2 per cohort) received placebo. All 30 participants completed the study in accordance with the protocol, and all were included in the safety population. All 20 participants receiving ASP5354 were included in the PK population. Within each cohort and overall, 50% of participants were female (Table S1). The overall mean age was 43 (standard deviation, 11.3 years), mean BMI was 27.3 kg/m^2^ (standard deviation, 2.96), and 66.7% were white. Mean age and BMI were similar across all cohorts.

### Safety and Tolerability

Overall, treatment‐emergent AEs (TEAEs) during the study were reported in 3 participants (15.0%) who received ASP5354 and 2 participants (20.0%) who received placebo. Infusions did not lead to histamine release or hypersensitivity. Among participants receiving ASP5354, 1 receiving the 0.5‐mg dose experienced oral herpes and presyncope (both grade 1), 1 receiving the 8‐mg dose experienced a grade 1 urinary tract infection, and 1 receiving the 24‐mg dose experienced grade 1 headache, grade 2 dysuria, and grade 3 pyelonephritis (Table 2). No TEAE was considered by investigators to be related or possibly related to ASP5354. Participants who experienced oral herpes, urinary tract infection, and pyelonephritis received corrective medication.

**Table 2:** Treatment‐Emergent Adverse Events

		ASP5354					
	Placebo (n = 10)	0.1 mg (n = 4)	0.5 mg (n = 4)	2 mg (n = 4)	8 mg (n = 4)	24 mg (n = 4)	Overall^a^ (N = 20)
Total, n (%)	2 (20.0)	0	1 (25.0)	0	1 (25.0)	1 (25.0)	3 (15.0)
Oral herpes	0	0	1 (25.0)	0	0	0	1 (5.0)
Pyelonephritis	0	0	0	0	0	1 (25.0)	1 (5.0)
Urinary tract infection	0	0	0	0	1 (25.0)	0	1 (5.0)
Headache	0	0	0	0	0	1 (25.0)	1 (5.0)
Presyncope	0	0	1 (25.0)	0	0	0	1 (5.0)
Dysuria	0	0	0	0	0	1 (25.0)	1 (5.0)
Incontinence	1 (10.0)	0	0	0	0	0	0
Nausea	1 (10.0)	0	0	0	0	0	0
Vomiting	1 (10.0)	0	0	0	0	0	0

^a^ In participants who received ASP5354 (any dose).

The case of grade 3 pyelonephritis was a serious AE that began on day 2 following ASP5354 administration and was attributed to the urethral catheter. This participant was admitted to the hospital on day 3 and treated with ceftriaxone. On day 6, the participant was discharged after switching to oral Bactrim, pyridium, and ibuprofen, at which point the serious AE was considered resolved. There were no clinically significant results or trends in serum chemistry data, hematology data, vital sign measurements, ECG parameters, or physical examinations, nor were there any withdrawals from the study due to AEs or on‐study deaths.

### Pharmacokinetics

**Figure 2:**
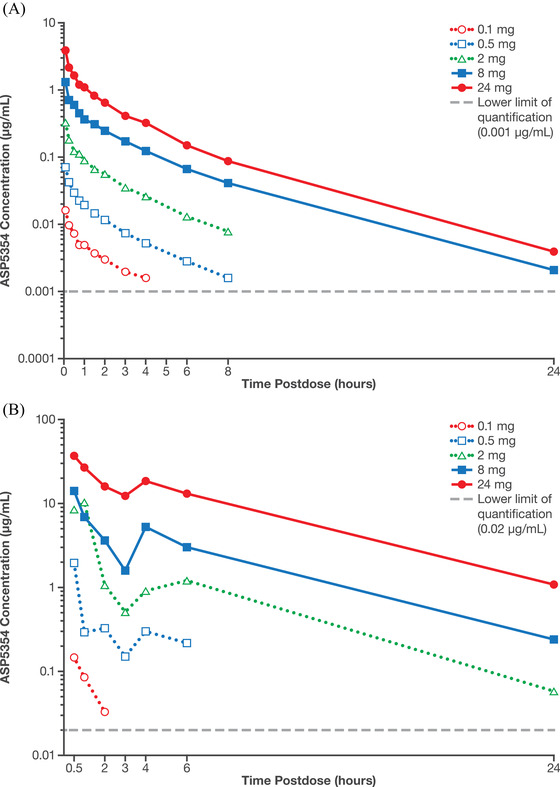
Arithmetic mean (A) plasma and (B) point urine concentration profiles of ASP5354 by dose.

**Figure 3:**
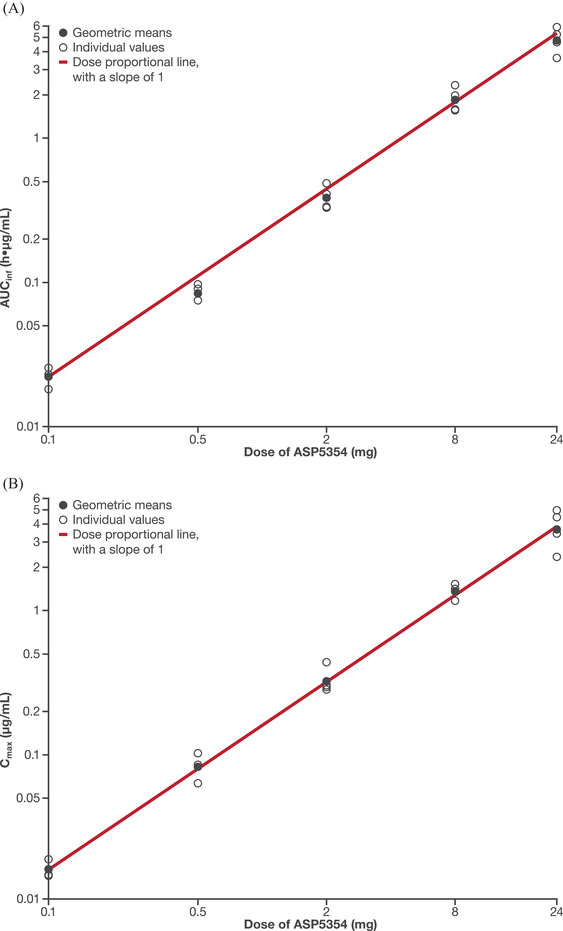
Dose‐proportionality assessment for ASP5354 parameters of (A) AUC_inf_ and (B) C_max_. AUC_inf_, area under the plasma concentration‐time curve from time 0 to infinity—logarithmic scale; C_max_, maximum observed plasma concentration.

ASP5354 arithmetic mean plasma concentrations are shown in Figure 2A. The mean terminal half‐life of ASP5354 ranged from 2.1 to 3.6 hours, and total body clearance and terminal volume of distribution were consistent across the dose range (Table 3). At doses ranging from 0.1 to 24 mg, increases in ASP5354 AUC_inf_ (Figure 3A) and maximum observed plasma concentration (Figure 3B) were approximately proportional to increases in dose. These dose‐proportional increases in exposure coupled with consistent half‐life and total body clearance estimates indicate linear PK for ASP5354 across the evaluated dose range.

**Table 3:** Summary of Plasma and Urine PK Parameters for ASP5354 Following Single IV Bolus Doses

	ASP5354				
	0.1 mg (n = 4)	0.5 mg (n = 4)	2 mg (n = 4)	8 mg (n = 4)	24 mg (n = 4)
Key Urine PK Parameters					
Ae_last_, mg	0.0768 (0.0214)	0.403 (0.0128)	1.68 (0.171)	8.01 (0.337)	23.1 (1.09)
Ae_last%_, %	76.8 (21.4)	80.6 (2.56)	84.1 (8.55)	100 (4.21)	96.3 (4.53)
CL_R_, L/h	3.57 (1.44)	4.85 (0.626)	4.45 (1.18)	4.43 (0.973)	4.92 (1.01)
Key Plasma PK Parameters					
AUC_0‐24_, μg · /mL	0.0223 (0.00309)	0.0842 (0.0112)	0.392 (0.0733)	1.87 (0.363)	4.85 (0.981)
AUC_inf_, μg · /mL	0.0224 (0.00310)	0.0843 (0.0112)	0.392 (0.0734)	1.88 (0.370)	4.87 (0.985)
AUC_inf_ (%extrap)^a^ (%)	19.7 (18.1‐25.7)	6.29 (4.63‐7.58)	5.86 (4.65‐7.78)	0.492 (0.327‐0.921)	0.375 (0.144‐0.770)
AUC_last_, μg · /mL	0.0178 (0.00313)	0.0791 (0.0110)	0.369 (0.0674)	1.87 (0.363)	4.85 (0.981)
C_max,_ μg/mL	0.0161 (0.00201)	0.0841 (0.0199)^b^	0.329 (0.0727)	1.37 (0.151)	3.81 (1.16)
C_0_, μg/mL	0.0223 (0.00235)	0.115 (0.0330)^b^	0.435 (0.108)	1.85 (0.244)	4.74 (1.56)
t_max_ ^a^, h	0.08 (0.08‐0.17)	0.08 (0.08‐0.08)	0.08 (0.08‐0.08)	0.08 (0.08‐0.08)	0.08 (0.08‐0.08)
t_last_ ^a^, h	4.00 (4.00‐6.00)	8.00 (8.00‐8.00)	8.00 (8.00‐8.02)	24.00 (24.00‐24.00)	24.03 (24.00‐24.03)
t_1/2_, h	2.24 (0.380)	2.23 (0.0988)	2.10 (0.147)	3.58 (0.245)	3.41 (0.420)
CL_tot_, L/h	4.54 (0.687)	6.01 (0.789)	5.22 (0.918)	4.38 (0.820)	5.10 (1.12)
V_z_, L	14.6 (2.70)	19.3 (2.79)	15.8 (2.28)	22.4 (2.95)	25.1 (6.51)

Arithmetic means (standard deviation) are presented unless otherwise noted.

Ae_last_, amount of unchanged drug excreted into the urine from time 0 to the time of the last quantifiable concentration; Ae_last%_, percentage of unchanged drug excreted into the urine from time 0 to the time of the last quantifiable concentration; AUC_0‐24_, area under the plasma concentration–time curve from time 0 to 24 h after dosing; AUC_inf_, area under the plasma concentration–time curve from time 0 to infinity; AUC_inf_(%extrap), area under the plasma concentration–time curve extrapolated from time t_last_ to infinity as a percentage of total area under the plasma concentration‐time curve; AUC_last_, area under the plasma concentration–time curve from time 0 to the time of the last quantifiable concentration; C_0_, back‐extrapolated plasma concentration at time zero; CL_R_, renal clearance; CL_tot_, total body clearance of drug from plasma; C_max_, maximum observed plasma concentration; IV, intravenous; PK, pharmacokinetic; SD, standard deviation; t_1/2_, apparent terminal plasma elimination half‐life; t_last_, time of last observed plasma concentration; t_max_, time of maximum observed plasma concentration; V_z_, volume of distribution during the terminal phase.

^a^ Median (minimum‐maximum).

^b^ n = 3.

Following IV administration, ASP5354 appeared rapidly in the urine, with quantifiable concentrations observed for all participants at the first postdose sample collection point (0.5 hours). Urinary ASP5354 concentrations were quantifiable for up to 6 hours after dosing in the 0.1‐mg and 0.5‐mg cohorts, and for up to 24 hours after dosing in the 2‐, 8‐, and 24‐mg cohorts (Figure 2B). Across all dose ranges, urinary excretion was nearly complete by 24 hours. The mean amount of intact ASP5354 recovered in urine was 0.0768, 0.403, 1.68, 8.01, and 23.1 mg following respective ASP5354 doses of 0.1, 0.5, 2, 8, or 24 mg. The corresponding percentage of the administered ASP5354 dose recovered unchanged in urine (percentage of Ae from time 0 to the time of last quantifiable concentration) ranged from 76.8% to 100% (Table 3). As a point of reference, in the preclinical study using a minipig model, ASP5354 was totally excreted (95%) within 6 hours after IV administration.^22^

Visible green urine coloration occurred in participants who received 8‐ and 24‐mg doses of ASP5354, but not in those receiving placebo. In 7 of these 8 participants, green urine coloration was first observed at the initial urine interval collection (0‐0.5 hours) (Table 4). In the remaining participant (8‐mg cohort), green urine coloration first appeared in the 2‐ to 2.5‐hour interval. Green urine was observed until 3 to 3.5 hours after dosing in 3 of the 4 participants in the 8‐mg cohort, and until 12 to 24 hours after dosing in 2 of the 4 participants in the 24‐mg cohort. In all cases, green urine coloration was no longer observed by 24 hours after dosing.

**Table 4:** Timing of Green Urine Coloration

Cohort	Order of Dosing per Cohort	Sex	Interval When Green Coloration Was First Observed, Hour Postdose	Interval When Green Coloration Was No Longer Observed, Hour Postdose
8 mg	1	Male	0‐0.5	1‐1.5
	2	Male	0‐0.5	3‐3.5
	3	Female	2‐2.5	3‐3.5
	4	Female	0‐0.5	3‐3.5
24 mg	1	Male	0‐0.5	8‐12
	2	Female	0‐0.5	8‐12
	3	Female	0‐0.5	12‐24
	4	Male	0‐0.5	12‐24

## Discussion

There are currently no FDA‐approved agents to facilitate intraoperative NIRF visualization of the ureter. Experimental studies of the dyes methylene blue and indocyanine green, which are approved for use in other indications,^23^, ^24^, ^25^ have revealed deficiencies in optical properties and route of administration and clearance, respectively, that make them unsuitable for such procedures.^12^ To address this unmet need, novel NIRF agents are being developed. Several of these, including IS‐001,^17^ IRDye 800‐BK,^26^, ^27^ and ZW800‐1,^28^ have recently progressed to first‐in‐human studies.

Here, we report first‐in‐human data for ASP5354, which was designed for high water solubility, low self‐aggregation (which can reduce fluorescence), and high optical and chemical stability.^20^ These results, obtained in healthy volunteers, provide key information regarding the safety and PK profiles of ASP5354, including a dose estimation for a phase 2 study design. Single ascending IV bolus doses of up to 24 mg did not lead to any ASP5354‐related TEAEs and there were no withdrawals due to AEs. One serious TEAE, grade 3 pyelonephritis, occurred in 1 participant and, consistent with urology observation spanning decades,^29^, ^30^ was found to be a complication related to the urethral catheter. No significant trends in serum chemistry data, hematology data, vital sign measurements, ECG parameters, or physical examinations were observed in any participant.

PK analysis revealed that ASP5354 is primarily excreted unchanged into urine, appearing there rapidly, with its excretion nearly complete at 24 hours. A single 8‐ or 24‐mg dose afforded noticeable green urine coloration for at least 3 hours in most participants. Linear and dose‐proportional ASP5354 plasma PK were observed across the evaluated dose range.

In conclusion, based on preclinical results demonstrating that IV ASP5354 allows distinct NIRF ureter visualization at 0.01 mg/kg,^22^ the ASP5354 urine concentrations and PK parameters found in this study support intraoperative NIRF ureter visualization using ASP5354 within the 0.1‐ to 24‐mg dose range. Collectively, these safety and PK results support further evaluation of ASP5354 for ureter detection during surgical procedures of the abdomen and pelvis.

## Conflicts of Interest

L.G., S.F., V.M., and B.G. are employees of Labcorp Drug Development Inc., which received funding from Astellas Pharma, Inc. T.M., M.T., and A.S. are employees of Astellas Pharma, Inc.

## Funding

This study was funded by Astellas Pharma, Inc.

## Supporting information

Supporting Information

Supporting Information
