# Supplementary material for: Randomized, Double‐Blind, Controlled Study to Evaluate Safety and Pharmacokinetics of Single Ascending Doses of ASP5354, an Investigational Imaging Product, in Healthy Adult Volunteers
Source: Clin Pharmacol Drug Dev. 2021 Aug 23;10(12):1460–8. doi: 10.1002/cpdd.1013 (PMC9292347; doi:10.1002/cpdd.1013)
Supplement: Supplementary file 1 — Supporting Information [file CPDD-10-1460-s002.docx]

**Supplemental On-Line Digital Content**

**METHODS**

**Rationale for dose levels**

The starting dose was 0.1 mg per subject. The rationale for the starting dose was based on the results of the toxicology studies in cynomolgus monkeys and the estimated clinical efficacious dose in humans.

The estimated clinical efficacious dose is 0.5 mg per subject. In the ex vivo imaging study of isolated pig ureter using ASP5354 chloride as a NIR-F agent,^1^ sufficient ureteral visualization was defined as the ureter was sufficiently noted visually in the captured images under fluorescent imaging at 1 μg/mL. In addition, in the imaging study of minipigs,^1^ the ureter was visually identifiable under fluorescent imaging at a urinary concentration greater than 1 μg/mL at 3 hours after intravenous administration of 0.01 mg/kg ASP5354. These observations support the clinical efficacious dose chosen in the study, permitting sufficient intraoperative visualization of the ureter for up to 3 hours, which exceeds the typical length of a routine surgical procedure (approximately 2 hours). Therefore, the estimated clinical efficacious dose was calculated as the dose that gives urine concentration above 1 μg/mL at 3 hours after intravenous administration of ASP5354 with body-weight correction using the following equation:

Estimated clinical efficacious dose = 0.01 mg/kg (animal dose) x (40 kg [animal weight] / 60 kg [human weight])^0.33^ x 60 kg (human weight) = 0.5 mg/subject

**Criteria for handling concentrations below the limit of quantification in pharmacokinetic analysis**

Concentration values that were below the level of quantification (BLQ) were set to zero, with defined exceptions as follows:

- Any embedded BLQ value (between two quantifiable concentrations) and BLQ values following the last quantifiable concentration in a profile were set to missing for the purposes of pharmacokinetic (PK) analysis.
- If there were late positive concentration values following two BLQ concentration values in the apparent terminal phase, these values were evaluated. If these values were considered to be anomalous, they were set to missing.
- If an entire concentration-time profile was BLQ, the profile was excluded from the PK analysis.
- If a predose concentration was missing, these values were set to zero by default in Phoenix WinNonlin.

**Bioanalysis of ASP5354**

All analytical procedures associated with this sample analysis were conducted with a method validated in CMIC Pharma Science Co., Ltd.

*Determination of ASP5354 in Human Urine Samples*

- 1. **Reference Standards**
     1. **ASP5354 Chloride (AS3315354-CL)**

Molecular weight 3079.44 (salt) 3043.99 (free form)

Supplier Astellas Analytical Science Laboratories, Inc.

(Kyoto, Japan)

Lot No. 1001188G

Conversion factor to free form 1.012

Purity 91.0%

Conversion factor to purity 1.099

Total correction factor (salt, purity) 1.112

Storage conditions Store in a freezer (−80°C, actual temperature:

–81.5°C to –74.6°C), Protection from light, Filled with nitrogen gas

Usage Relative humidity of 10% or lower

- - 1. **AS3366010-CL (IS; D_12_-ASP5354 Chloride)**

Supplier Astellas Pharma Inc. (Ibaraki, Japan)

Lot No. L74627226

Chemical purity 81.9%

Correction factor (purity) Regarded as 1.000

Storage conditions Store in a freezer (−80°C, actual temperature:

–83.3°C to –73.5°C), Protection from light, Filled with nitrogen gas

Usage Relative humidity of 10% or lower

- - 1. **AS3331000-CL (Synthetic Intermediate, Used as a Reagent)**

Supplier Astellas Pharma Inc. (Ibaraki, Japan)

Lot No. 151201

Purity 99%

Correction factor (purity) Regarded as 1.000

Storage conditions Store in a refrigerator (2°C to 8°C, actual temperature: 4.8°C to 6.9), Protection from light, Store with desiccant

- 1. **Matrix**

The following blank human urine was used in the analytical runs of this sample analysis study:

Supplier BizCom Japan, Inc. (Tokyo, Japan)

Imported from BioIVT (Westbury, NY, USA)

Lot Number Individual urine:

Male; BRH1518278, BRH1518279, BRH1518280

Female; BRH1518281, BRH1518282, BRH1518283

Storage Conditions Store in a freezer (acceptable range of freezer: −70°C or

below, actual temperature: –88.1°C to –70.1°C)

Blank human urine containing 0.1% Tween20 was used for this study. Pooled blank urine containing 0.1% Tween20 was prepared from three male donors and three female donors.

- 1. **Study Samples Receipt and Inventory**

A summary of sample receipt and inventory is provided below. Detailed listings were kept in the study file.

| Sample Origins | Labcorp Drug Development (formerly Covance) CRU Inc.  1900 Mason Ave. Suite 140 Daytona Beach, FL, 32117, USA |
| --- | --- |
| Collection Dates (first – last) | 20 November 2018 and 04 May 2019 |
| Additive | 0.1% Tween20 (final concentration in urine) |
| Receipt Dates (first – last) | 30 November 2018 and 25 June 2019 |
| Storage (nominal) | −80°C (actual temperature: −88.1°C to −70.1°C) |
| Total number of samples per protocol | 600 |
| Total number of samples received^1)^ | 600 |
| Total number of samples analyzed | 400 |
| Total number of samples not analyzed  (i.e. placebo^2)^, comparator(s)) | 200 |
| Experimental dates (first – last) | 04 December 2018 and 30 May 2019 |
| Long-term frozen stability | 364 days at −80°C (−70°C or below)  All samples analyzed within established stability period. |

1. Back-up samples (599 samples) were collected. Back-up sample of one point was not obtained.
2. Placebo dose group samples were not analyzed.

The number of samples per subject are specified in the following.

| **Urine (20 samples/subject)** | | |
| --- | --- | --- |
| **Part/Period** | **Day/visit** | **Time points** |
| Cohort 1 to 5 | Day 1 to 2 | 0.5, 1, 2, 3, 4, 6 and 24 hours postdose |
|  | **Day/visit** | **Time interval** |
|  | Day 1 to 2 | -1 to 0, 0 to 0.5, 0.5 to 1, 1 to 1.5, 1.5 to 2, 2 to 2.5, 2.5 to 3, 3 to 3.5,  3.5 to 4, 4 to 6, 6 to 8, 8 to 12 and 12 to 24 hours postdose |

- 1. **Sample Analysis Run Description**

Each run included a reagent blank, a matrix blank, a control zero (matrix blank containing only IS), calibration samples (n=1 for each concentration), and QC samples (LQC, MQC, and HQC, n=2 for each concentration). Study samples were bracketed by QC samples. When possible, study samples from a subject were analyzed in the same analytical run. Samples were analyzed in single replicate. At least one blank sample was placed immediately following a ULOQ (the highest calibration) sample to monitor carryover.

- 1. **Chemicals, Solvents, and Consumables**

| **Chemicals/Solvents/Consumables** | **Grade/Type** | **Manufacturer** |
| --- | --- | --- |
| Acetonitrile | HPLC grade | FUJIFILM Wako Pure Chemical (Osaka, Japan) |
| Methanol | HPLC grade | FUJIFILM Wako Pure Chemical (Osaka, Japan) |
| Formic acid | Guaranteed reagent | FUJIFILM Wako Pure Chemical (Osaka, Japan) |
| Tween20 (Polyoxyethylene (20) sorbitan monolaurate) | For Biochemistry | FUJIFILM Wako Pure Chemical (Osaka, Japan) |
| Water | NA | NA |
| Volumetric flask (Low actinic) | PYREX^®^ | AGC TECHNO GLASS  (Shizuoka, Japan) |
| Cylinder | PYREX^®^ | AGC TECHNO GLASS  (Shizuoka, Japan) |
| Transfer pipette | PYREX^®^ | AGC TECHNO GLASS  (Shizuoka, Japan) |
| Safe-Lock Tube  (described as “micro tube” hereafter) | 1.5 mL | Eppendorf (Hamburg, Germany) |
| OASIS HLB µElution Plate | 30 µm | Waters (Milford, MA, USA) |
| 96-well Sample Collection Plate (described as “1 mL plate” hereafter) | 1 mL | Waters (Milford, MA, USA) |
| HPLC vial (polypropylene vial) | 250 µL | Agilent Technologies (Santa Clara, CA, USA) |
| CAPCELL PAK ADME  (2.1 mm I.D. × 20 mm, particle size 2 µm) | NA | OSAKA SODA (Osaka, Japan) |

Note: Water was purified with an ultrapure water system.

- 1. **Instrumentation and Equipment**

| **Instrumentation/Equipment** | **Model** | **Manufacturer** |
| --- | --- | --- |
| LC-MS/MS system | | |
| HPLC system | NANOSPACE NASCA 2  system (No. 15082) | OSAKA SODA (Osaka,  Japan) |
| Mass spectrometer | QTRAP6500, equipped with TurboIonSpray interface (No. 2) | SCIEX (Framingham, MA, USA) |
| Eppendorf pipette | 4910 | Eppendorf (Hamburg, Germany) |
| Multipette | E3 | Eppendorf (Hamburg, Germany) |
| Electronic semi-micro balance | ME235S | Sartorius (Goettingen, Germany) |
| Refrigerated centrifuge | H-80R | Kokusan (Tokyo, Japan) |
| High speed refrigerated micro centrifuge | MX-300, MX-301 | Tomy Seiko (Tokyo, Japan) |
| Ultrapure water system | Milli-Q Integral system | Merck (Darmstadt, Germany) |
| Handy type temperature and humidity meter | HP22-A | Rotronic (Bassersdorf, Switzerland) |
| Eppendorf MixMate | NA | Eppendorf (Hamburg, Germany) |

- 1. **Preparation of Reagent Solutions**
     1. **1 mg/mL Synthetic Intermediate**

AS3331000-CL (1.51 mg or 1.27 mg) was weighed and dissolved in water / acetonitrile (1:1, v/v) to obtain a nominal concentration of 1 mg/mL. Preparation was done under the yellow light. The solution was stored in a refrigerator at 2°C to 8°C and used within 3 months.

[Volume of water / acetonitrile (1:1, v/v) (mL) = actual weighed value (mg) × 1 (mL)]

- - 1. **20 µg/mL Synthetic Intermediate**

Water / acetonitrile (1:1, v/v) and 1 mg/mL synthetic intermediate were mixed at a ratio of 980:20 (v/v). Preparation was done under the yellow light. The solution was prepared at the time of use, and not stored.

- - 1. **Water / Formic Acid (100:0.1, v/v)**

Water and formic acid were mixed at a ratio of 100:0.1 (v/v). The solution was stored in a refrigerator at 2°C to 8°C and used within 3 months.

- - 1. **Water / Methanol (9:1, v/v)**

Water and methanol were mixed at a ratio of 9:1 (v/v). The solution was stored at room temperature and used within 3 months.

- - 1. **Water / Acetonitrile (1:1, v/v)**

Water and acetonitrile were mixed at a ratio of 1:1 (v/v). The solution was stored at room temperature and used within 3 months.

- - 1. **Water / Formic Acid (100:0.1, v/v) (Mobile Phase A)**

Water and formic acid were mixed at a ratio of 100:0.1 (v/v). The solution was stored at room temperature and used within 1 week.

- - 1. **Acetonitrile / Formic Acid (100:0.1, v/v) (Mobile Phase B)**

Acetonitrile and formic acid were mixed at a ratio of 100:0.1 (v/v). The solution was stored at room temperature and used within 1 week.

- - 1. **Water / Acetonitrile / Formic Acid (50:50:0.1, v/v/v) (Wash Solvent)**

Water, acetonitrile and formic acid were mixed at a ratio of 50:50:0.1 (v/v/v). The solution was stored at room temperature and used within 1 month.

- - 1. **10% Tween20**

Water and Tween20 were mixed gently at a ratio of 9:1 (v/v). The solution was prepared at the time of use, and not stored.

- - 1. **Urine Containing 0.1% Tween20**

To blank urine, 10% Tween20 was added at a rate of 10 µL per 1 mL urine, and mixed. The urine containing 0.1% Tween20 was stored at −70°C or below and used within one year from urine receipt date.

Note: Reagents prepared in another study (Study No. CB181708) were used as occasion demands.

- 1. **Preparation of Standard Stock Solutions**

All preparation was done under the yellow light.

Standard stock solutions prepared in another study (Study No. CB181708) were used.

Calibration samples and QC samples were prepared from separate stock solutions. All stock solutions were stored in amber glass vials in a refrigerator at 2°C to 8°C (actual temperature: 3.5°C to 6.6°C) and protected from light. The stock solutions were stored for a period of at maximum 84 days.

- - 1. **Calibration Stock Solution [A]**

ASP5354 chloride (approximately 11.1 mg*^1^, acceptable range: 10.0 mg to 12.2 mg, actual value: 11.02 mg and 10.69 mg) was accurately weighed and dissolved in water / acetonitrile (1:1, v/v) to obtain a nominal concentration of 2000 µg/mL.

*^1^: Target weight (mg)

= 10.0 (mg) ×1.112 (total correction factor)

Volume of water / acetonitrile (1:1, v/v) (mL)

= [actual weighed value (mg) / 11.1 (mg)] × 5 (mL)

- - 1. **QC Stock Solution [QA]**

ASP5354 chloride (approximately 11.1 mg*^1^, acceptable range: 10.0 mg to 12.2 mg, actual value: 10.67 mg and 10.08 mg) was accurately weighed and dissolved in water / acetonitrile (1:1, v/v) to obtain a nominal concentration of 2000 µg/mL.

*^1^: Target weight (mg)

= 10.0 (mg) ×1.112 (total correction factor)

Volume of water / acetonitrile (1:1, v/v) (mL)

= [actual weighed value (mg) / 11.1 (mg)] × 5 (mL)

- 1. **Preparation of Standard Working Solutions**

All preparation was done under the yellow light.

- - 1. **Calibration Working Solutions**

The calibration stock solution was serially diluted with water / acetonitrile (1:1, v/v) to prepare the following calibration working solutions as indicated in the table below.

The calibration working solutions of S4 to S10 were divided into 1 mL aliquots and stored. After use of the stored calibration working solution, the remaining solution was discarded and not re-stored. These solutions were stored in amber glass vials in a refrigerator at 2°C to 8°C (actual temperature: 3.5°C to 6.6°C) and protected from light. The working solutions were stored for a period of at maximum 12 days.

The calibration working solutions of S1 to S3 were prepared at the time of use, and not stored.

| **Working Solution ID** | **Source Solution ID** | **Concentration of Source Solution (µg/mL)** | **Volume of Source Solution Aliquoted**  **(mL)** | **Final Volume of Working Solution**  **(mL)** | **Final Concentration of Working Solution**  **(µg/mL)** |
| --- | --- | --- | --- | --- | --- |
| S10 | A | 2000 | 0.08 | 20 | 8.00 |
| S9 | A | 2000 | 0.072 | 20 | 7.20 |
| S8 | A | 2000 | 0.04 | 20 | 4.00 |
| S7 | S10 | 8.00 | 2.5 | 10 | 2.00 |
| S6 | S10 | 8.00 | 1 | 10 | 0.800 |
| S5 | S8 | 4.00 | 1 | 10 | 0.400 |
| S4 | S7 | 2.00 | 1 | 10 | 0.200 |
| S3 | S6 | 0.800 | 0.5 | 5 | 0.0800 |
| S2 | S5 | 0.400 | 0.5 | 5 | 0.0400 |
| S1 | S4 | 0.200 | 0.5 | 5 | 0.0200 |

- - 1. **QC Working Solutions**

The QC stock solution was serially diluted with water / acetonitrile (1:1, v/v) to prepare the following QC working solutions as indicated in the table below.

These QC working solutions were divided into 1 mL aliquots and stored. After use of the stored QC working solution, the remaining solution was discarded and not be re-stored.

These solutions were stored in amber glass vials in a refrigerator at 2°C to 8°C (actual temperature: 4.6°C to 6.2°C) and protected from light. The working solutions were stored for a period of at maximum 4 days.

| **Working Solution ID** | **Source Solution ID** | **Concentrations of Source Solution (µg/mL)** | **Volume of Source Solution Aliquoted**  **(mL)** | **Final Volume of Working Solution**  **(mL)** | **Final Concentration of Working Solution**  **(µg/mL)** |
| --- | --- | --- | --- | --- | --- |
| Q3 | QA | 2000 | 1.6 | 5 | 640 |
| Q2 | QA | 2000 | 0.15 | 5 | 60.0 |
| Q1 | Q2 | 60.0 | 1 | 10 | 6.00 |

- 1. **Preparation of IS Solutions**

All preparation was done under the yellow light.

IS stock solution prepared in another study (Study No. CB181708) was used.

- - 1. **IS Stock Solution [IA]**

AS3366010-CL (IS) (approximately 10.0 mg, acceptable range: 9.0 mg to 11.0 mg, actual value: 10.13 mg and 10.35 mg) was accurately weighed and dissolved in water / acetonitrile (1:1, v/v) to obtain a nominal concentration of 1000 µg/mL. The IS stock solutions were used immediately after preparation.

Volume of water / acetonitrile (1:1, v/v) (mL)

= [actual weighed value (mg) / 10.0 (mg)] × 10 (mL)

- - 1. **IS Working Solutions**

The IS stock solution was serially diluted with water / acetonitrile (1:1, v/v) to prepare the following IS working solutions as indicated in the table below.

When storing, the IS working solutions were divided into 1 mL aliquots and stored in amber glass vials. After use of the stored IS working solution, the remaining solution was discarded and not be re-stored. These solutions were stored in amber glass vials in a refrigerator at 2°C to 8°C (actual temperature: 3.5°C to 6.6°C) and protected from light. IS2 was stored for a period of at maximum 68 days. IS1 was stored for a period of at maximum 26 days.

| **Working Solution ID** | **Source Solution ID** | **Concentrations of Source Solution (µg/mL)** | **Volume of Source Solution Aliquoted**  **(µL)** | **Final Volume of Working Solution**  **(mL)** | **Final Concentration of Working Solution**  **(µg/mL)** |
| --- | --- | --- | --- | --- | --- |
| IS2 | IA | 1000 | 1000 | 10 | 100 |
| IS1 | IS2 | 100 | 30 | 10 | 0.300 |

- 1. **Preparation of Calibration and QC Samples**

Calibration and QC sample were prepared in the same matrix and anticoagulant as expected for study samples.

- - 1. **Preparation of Calibration Samples**

Preparation of calibration standards was performed in ice water bath.

The calibration samples were freshly prepared on the day of use by adding 50 µL of 20 µg/mL synthetic intermediate, 400 µL of water / formic acid (100:0.1, v/v), and 5 µL each of the individual calibration working solutions to of 5 µL of pooled blank urine containing 0.1% Tween20 to prepare 20.0, 40.0, 80.0, 200, 400, 800, 2000, 4000, 7200, and 8000 ng/mL

of ASP5354 in urine.

| **Calibration Sample**  **ID** | **Source Solution ID** | **Concentration of Source Solution (µg/mL)** | **Volume of Source Solution Aliquoted**  **(µL)** | **Volume of Blank Urine containing 0.1% Tween20**  **(µL)** | **Calibration Sample Concentration (ng/mL)** |
| --- | --- | --- | --- | --- | --- |
| STD1 | S1 | 0.0200 | 5 | 5 | 20.0 |
| STD2 | S2 | 0.0400 | 5 | 5 | 40.0 |
| STD3 | S3 | 0.0800 | 5 | 5 | 80.0 |
| STD4 | S4 | 0.200 | 5 | 5 | 200 |
| STD5 | S5 | 0.400 | 5 | 5 | 400 |
| STD6 | S6 | 0.800 | 5 | 5 | 800 |
| STD7 | S7 | 2.00 | 5 | 5 | 2000 |
| STD8 | S8 | 4.00 | 5 | 5 | 4000 |
| STD9 | S9 | 7.20 | 5 | 5 | 7200 |
| STD10 | S10 | 8.00 | 5 | 5 | 8000 |

- - 1. **Preparation of QC Samples**

Preparation of QC samples was performed in ice water bath.

The QC samples were prepared as bulk solutions. Pooled blank urine containing 0.1% Tween20 was put into separate micro tubes, followed by the addition of each QC working solution as indicated in the table below. The samples were mixed well. When the samples were stored, 100 µL portions of each QC samples were aliquoted into pre-labeled micro tubes.

All QC samples were stored in a freezer at −70°C or below (actual temperature: −86.1°C to – 70.1°C). The QC samples were stored for a period of at maximum 364 days.

| **QC**  **Sample ID** | **Source Solution ID** | **Concentration of Source Solution (µg/mL)** | **Volume of Source Solution Aliquoted**  **(µL)** | **Volume of Blank Urine containing 0.1% Tween20**  **(µL)** | **QC Sample Concentration (ng/mL)** |
| --- | --- | --- | --- | --- | --- |
| LQC | Q1 | 6.00 | 5 | 495 | 60.0 |
| MQC | Q2 | 60.0 | 5 | 495 | 600 |
| HQC | Q3 | 640 | 5 | 495 | 6400 |

- 1. **Dilution of Samples**

When the assay value of a study sample was expected to exceed or exceeded the highest concentration on the calibration curve, the sample was diluted 10 or 100-fold with pooled blank urine containing 0.1% Tween20 as indicated in the table below and measured.

| **Dilution Factor** | **First dilution** | | **Second dilution** | |
| --- | --- | --- | --- | --- |
|  | **Volume of Study**  **Sample (µL)** | **Volume of Blank Matrix (µL)** | **Volume of First Dilution Aliquoted (µL)** | **Volume of Blank Matrix (µL)** |
| 10 | 5 | 45 |  |  |
| 100 | 5 | 45 | 5 | 45 |

- 1. **Sample Pre-treatment**
     1. **Sample Pre-treatment Procedure**

Samples included contamination and carryover samples (reagent blank, matrix blank [matrix blank spiked without IS], and control zero [matrix blank spiked with IS]) with a set of calibration and QC samples were treated in accordance with the following procedures:

- - - - 5 µL of each parent aliquot of the pooled blank urine containing 0.1% Tween20, QC samples and study samples were added to each micro tube in ice water bath. For reagent blank, 5 µL of water was added in ice water bath.
      - 20 µg/mL Synthetic intermediate (50 µL) was added to each of the samples in ice water bath.
      - Water / formic acid (100:0.1, v/v) (400 µL) was added to each of the samples described above in ice water bath.
      - Calibration working solution (5 µL) was added to each calibration sample as described in Sec[tion 5.11.1](#_bookmark42) [Preparation of Calibration Samples](#_bookmark42) in ice water bath.
      - Water / acetonitrile (1:1, v/v) (15 µL) was added to the reagent blank and the matrix blank in ice water bath.
      - Water / acetonitrile (1:1, v/v) (5 µL) was added to the control zero, QC samples and study samples in ice water bath.
      - IS working solution [IS1 (0.300 µg/mL)] (10 µL) was added to each calibration sample, the control zero, QC samples and study samples in ice water bath.
      - All tubes were mixed with an Eppendorf MixMate (preset value: 1500 rpm, 1 minute) and centrifuged at 20000×*g* for 10 minutes set at 4°C.
      - Solid-phase extraction plate (OASIS HLB µElution plate) was preconditioned successively with methanol (200 µL) and water (200 µL).
      - Each supernatant was applied to each well of the OASIS HLB µElution plate.
      - After washing with water / methanol (9:1, v/v) (200 µL) by centrifugation, the analyte and the IS were eluted with methanol (25 µL × 2) by centrifugation into a 1 mL plate added water (50 µL) (preset value of centrifugation: 200×*g*, 2 minutes, 4°C).
      - Each mixture was shaken with an Eppendorf MixMate (preset value: 1000 rpm, 30 seconds).
      - Transfer into HPLC vial.
      - A 12-µL portion of the solution was injected into the LC-MS/MS system.
    1. **Flow Chart of Sample Pre-treatment Procedure**

The following flow chart shows the sample treatment procedure:

|  | | In ice water bath | | | | | |
| --- | --- | --- | --- | --- | --- | --- | --- |
|  | | Starting material | *1 (µL) | *2 (µL) | Calibration working solution (µL) | Water / acetonitrile (1:1, v/v) (µL) | IS working solution (IS1) (µL) |
| Blank | | Pooled blank urine containing 0.1% Tween20 (5 µL) or water (5 µL) | 50 | 400 | — | 15 | — |
| Calibration samples | | Pooled blank urine containing 0.1%  Tween20 (5 µL) | 50 | 400 | 5 | — | 10 |
| Control zero | | Pooled blank urine containing 0.1%  Tween20 (5 µL) | 50 | 400 | — | 5 | 10 |
| QC  samples | | QC sample (5 µL) | 50 | 400 | — | 5 | 10 |
| Study samples | | Study sample (5 µL) | 50 | 400 | — | 5 | 10 |
|  | *1: 20 µg/mL Synthetic intermediate  *2: Water / formic acid (100:0.1, v/v) | | | | | | |

1. Mix with an Eppendorf MixMate (preset value: 1500 rpm, 1 minute)
2. Centrifuge (preset value: 20000×*g*, 10 minutes, 4°C)
3. Load supernatant onto OASIS HLB µElution plate
   1. Preconditioned with methanol (200 µL) and water (200 µL)
4. Wash: water / methanol (9:1, v/v) (200 µL)
   1. (by centrifugation, preset value: 200×*g*, 2 minutes, 4°C) Add water (50 µL) to a 1 mL plate
5. Elute to a 1 mL plate: methanol (25 µL×2)
   1. (by centrifugation, preset value: 200×*g*, 2 minutes, 4°C)
6. Shake with an Eppendorf MixMate (preset value: 1000 rpm, 30 seconds)
7. Transfer into HPLC vial
8. Inject into the LC-MS/MS system (12 µL)

- 1. **HPLC Conditions**

HPLC system: NANOSPACE NASCA 2 system (OSAKA SODA)

Analytical column: CAPCELL PAK ADME

(2.1 mm I.D. × 20 mm, particle size 2 µm) (OSAKA SODA)

Mobile phase A: Water / formic acid (100:0.1, v/v)

Mobile phase B: Acetonitrile / formic acid (100:0.1, v/v)

| Gradient: | Time | Flow Rate | Mobile Phase | Mobile Phase |
| --- | --- | --- | --- | --- |
|  | (min) | (µL/min) | B (%) | A (%) |
|  | Init | 200 | 25 | 75 |
|  | 8.8 | 200 | 40 | 60 |
|  | 8.9 | 400 | 90 | 10 |
|  | 9.4 | 400 | 90 | 10 |
|  | 9.5 | 400 | 25 | 75 |
|  | 11.0 | 400 | 25 | 75 |

Wash solvent: Water / acetonitrile / formic acid (50:50:0.1, v/v/v)

Column temperature: 60°C (preset temperature)

Autosampler temperature: 5°C (preset temperature)

Injection volume: 12 µL

Run time: 11.0 min

Retention time for ASP5354: 3.9 min (3.3 – 4.5 min)

Retention time for IS: 3.9 min (3.3 – 4.5 min)

- 1. **MS/MS Conditions**

Mass spectrometer: QTRAP6500 (SCIEX)

Software: Analyst 1.6.2 (SCIEX)

Ionization: ESI (Turbo ion spray)

Ion polarity: Positive

Scan mode: MRM

IonSpray voltage: 4500 V

Temperature (TEM): 400°C

Nebulizer gas (GS1): 50 psi

Turbo gas (GS2): 55 psi

Curtain gas (CUR) (nitrogen): 30 psi

Collision gas (CAD) (nitrogen): 12

Entrance potential (EP): 10 V

Valve position: Time (min) Valve Position

0.0 A (Divert)

2.0 B (MS)

6.0 A Divert)

Monitored ion, DP, CE, CXP and dwell time:

| Name | Precursor Ion | Product Ion | DP | CE | CXP | Dwell Time |
| --- | --- | --- | --- | --- | --- | --- |
|  | (*m/z*) | (*m/z*) | (V) | (V) | (V) | (msec) |
| ASP5354 | 1015 | 325 | 70 | 26 | 21 | 745 |
| IS | 1019 | 717 | 81 | 33 | 30 | 245 |

**Integration settings**

Integration algorithm: Intelliquan

- 1. **System Suitability**

System suitability was evaluated by injecting the standard solution at LLOQ level with the IS (n=6) to ensure the system was working properly prior to the start of an analytical run or a consecutive series of runs.

The retention times, peak shapes, and analyte and the IS background levels were evaluated for consistency.

The system was considered to be acceptable if the precision (CV) of the IS peak areas and the peak area ratios does not exceed 20.0%.

- - 1. **Preparation of System Suitability Check Sample**

The calibration working solution [S1 (0.0200 µg/mL); 5 µL], the IS working solution [IS1 (0.300 µg/mL); 10 µL], water (45 µL), and methanol (40 µL) were put into a micro tube to prepare a system suitability check sample for injection into LC-MS/MS system. A 12-µL portion of the solution was injected into LC-MS/MS system.

- 1. **Contamination and Carryover**

Samples to monitor contamination and carryover were incorporated into each analytical run.

Contamination during sample processing was investigated by injecting a reagent blank, a matrix blank and a control zero.

Carryover from the analytical equipment was investigated within each analytical run. The extent of the carryover was determined by injecting blank matrix samples (n=1) immediately after the ULOQ (the highest calibration) sample.

The LLOQ (the lowest calibration) sample in the same run was used as the reference. Contamination and carryover acceptance criteria are as follows:

1. The ratio of interference peaks versus the analyte peak at LLOQ has to be less than 0.200,
2. The ratio of interference peaks versus the IS peak has to be less than 0.0500.

*Determination of ASP5354 in Human Plasma Samples*

- 1. **Reference Standards**
     1. **ASP5354 Chloride (AS3315354-CL)**

Molecular weight 3079.44 (salt) 3043.99 (free form)

Supplier Astellas Analytical Science Laboratories, Inc. (Kyoto, Japan)

Lot No. 1001188G

Conversion factor to free form 1.012

Purity 91.0%

Conversion factor to purity 1.099

Total correction factor (salt, purity) 1.112

Storage conditions Store in a freezer (−80°C, actual temperature:

–81.5°C to –74.6°C), Protection from light, Filled with nitrogen gas

Usage Relative humidity of 10% or lower

- - 1. **AS3366010-CL (IS; D_12_-ASP5354 Chloride)**

Supplier Astellas Pharma Inc. (Ibaraki, Japan)

Lot No. L74627226

Chemical purity 81.9%

Correction factor (purity) Regarded as 1.000

Storage conditions Store in a freezer (−80°C, actual temperature:

–83.3°C to –73.5°C), Protection from light, Filled with nitrogen gas

Usage Relative humidity of 10% or lower

- 1. **Matrix**

The following blank human plasma was used in the analytical runs of this sample analysis study:

Anti-coagulant K_2_ EDTA

Supplier Kohjin Bio Co., Ltd. (Saitama, Japan)

Imported from Tennessee Blood Services (Memphis, TN, USA)

Lot Number Individual plasma:

Male; R500228, R513170, R513171

Female; R326022, R326023, R500063

Storage Conditions Store in a freezer (acceptable range of freezer: −70°C or

below, actual temperature: –88.1°C to –70.1°C)

Blank human matrix pools were prepared from three male donors and three female donors.

- 1. **Study Samples Receipt and Inventory**

A summary of sample receipt and inventory is provided below. Detailed listings were kept in the study file.

| Sample Origins | Labcorp Drug Development (formerly Covance) CRU Inc.  1900 Mason Ave. Suite 140 Daytona Beach, FL, 32117, USA |
| --- | --- |
| Collection Dates (first – last) | 20 November 2018 and 04 May 2019 |
| Anti-coagulant | K_2_ EDTA |
| Receipt Dates (first – last) | 30 November 2018 and 25 June 2019 |
| Storage (nominal) | −80°C (actual temperature: −88.1°C to −70.1°C) |
| Total number of samples per protocol | 390 |
| Total number of samples received^1)^ | 388 |
| Total number of samples analyzed | 259 |
| Total number of samples not analyzed (i.e. placebo^2)^, comparator(s)) | 129 |
| Experimental dates (first – last) | 03 December 2018 and 30 May 2019 |
| Long-term frozen stability | 364 days at −80°C (−70°C or below)  All samples analyzed within established stability period. |

1. An equal number of back-up samples were collected.
2. Placebo dose group samples were not analyzed.

Back-up samples of which original concentrations were clearly out of pharmacokinetics profiles were analyzed and reported ([Table 6)](#_bookmark78). The data of back-up samples were used only for comparison purposes and not for the final drug concentration results.

The number of samples per subject are specified in the following.

| **Plasma (13 samples/subject)** | | |
| --- | --- | --- |
| **Part/Period** | **Day/visit** | **Time points** |
| Cohort 1 to 5 | Day 1 to 2 | Predose, 5, 15, 30 and 45 minutes postdose and 1, 1.5, 2, 3, 4 ,6 ,8 and  24 hours postdose |

- 1. **Sample Analysis Run Description**

Each run included a reagent blank, a matrix blank, a control zero (matrix blank containing only IS), calibration samples (n=1 for each concentration), and QC samples (LQC, MQC, and HQC, n=2 for each concentration). Study samples were bracketed by QC samples. When possible, study samples from a subject were analyzed in the same analytical run. Samples were analyzed in single replicate. At least one blank sample was placed immediately following a ULOQ (the highest calibration) sample to monitor carryover.

- 1. **Chemicals, Solvents, and Consumables**

| **Chemicals/Solvents/Consumables** | **Grade/Type** | **Manufacturer** |
| --- | --- | --- |
| Acetonitrile | HPLC grade | FUJIFILM Wako Pure Chemical (Osaka, Japan) |
| Methanol | HPLC grade | FUJIFILM Wako Pure Chemical (Osaka, Japan) |
| Formic acid | Guaranteed reagent | FUJIFILM Wako Pure Chemical (Osaka, Japan) |
| Water | NA | NA |
| Volumetric flask (Low actinic) | PYREX^®^ | AGC TECHNO GLASS  (Shizuoka, Japan) |
| Cylinder | PYREX^®^ | AGC TECHNO GLASS  (Shizuoka, Japan) |
| Transfer pipette | PYREX^®^ | AGC TECHNO GLASS  (Shizuoka, Japan) |
| Safe-Lock Tube  (described as “micro tube” hereafter) | 1.5 mL | Eppendorf (Hamburg, Germany) |

| **Chemicals/Solvents/Consumables** | **Grade/Type** | **Manufacturer** |
| --- | --- | --- |
| Polypropylene conical tube (described as “PP tube” hereafter) | 15 mL | CORNING (Corning, NY, USA) |
| OASIS HLB µElution Plate | 30 µm | Waters (Milford, MA, USA) |
| 96-well Sample Collection Plate (described as “1 mL plate” hereafter) | 1 mL | Waters (Milford, MA, USA) |
| HPLC vial (polypropylene vial) | 250 µL | Agilent Technologies (Santa Clara, CA, USA) |
| CAPCELL PAK ADME  (2.1 mm I.D. × 20 mm, particle size 2 µm) | NA | OSAKA SODA (Osaka, Japan) |

Note: Water was purified with an ultrapure water system.

- 1. **Instrumentation and Equipment**

| **Instrumentation/Equipment** | **Model** | **Manufacturer** |
| --- | --- | --- |
| LC-MS/MS system | | |
| HPLC system | NANOSPACE NASCA 2  system (No. 15082) | OSAKA SODA (Osaka,  Japan) |
| Mass spectrometer | QTRAP6500, equipped with TurboIonSpray interface (No. 2) | SCIEX (Framingham, MA, USA) |
| Eppendorf pipette | 4910 | Eppendorf (Hamburg, Germany) |
| Multipette | E3 | Eppendorf (Hamburg, Germany) |
| Electronic semi-micro balance | ME235S | Sartorius (Goettingen, Germany) |
| Refrigerated centrifuge | H-80R | Kokusan (Tokyo, Japan) |

| **Instrumentation/Equipment** | **Model** | **Manufacturer** |
| --- | --- | --- |
| High speed refrigerated micro centrifuge | MX-300, MX-301 | Tomy Seiko (Tokyo, Japan) |
| Ultrapure water system | Milli-Q Integral system | Merck (Darmstadt, Germany) |
| Handy type temperature and humidity meter | HP22-A | Rotronic (Bassersdorf, Switzerland) |
| Eppendorf MixMate | NA | Eppendorf (Hamburg, Germany) |

- 1. **Preparation of Reagent Solutions**
     1. **Water / Formic Acid (100:0.1, v/v)**

Water and formic acid were mixed at a ratio of 100:0.1 (v/v). The solution was stored in a refrigerator at 2°C to 8°C and used within 3 months.

- - 1. **Water / Methanol (9:1, v/v)**

Water and methanol were mixed at a ratio of 9:1 (v/v). The solution was stored at room temperature and used within 3 months.

- - 1. **Water / Acetonitrile (1:1, v/v)**

Water and acetonitrile were mixed at a ratio of 1:1 (v/v). The solution was stored at room temperature and used within 3 months.

- - 1. **Water / Formic Acid (100:0.1, v/v) (Mobile Phase A)**

Water and formic acid were mixed at a ratio of 100:0.1 (v/v). The solution was stored at room temperature and used within 1 week.

- - 1. **Acetonitrile / Formic Acid (100:0.1, v/v) (Mobile Phase B)**

Acetonitrile and formic acid were mixed at a ratio of 100:0.1 (v/v). The solution was stored at room temperature and used within 1 week.

- - 1. **Water / Acetonitrile / Formic Acid (50:50:0.1, v/v/v) (Wash Solvent)**

Water, acetonitrile and formic acid were mixed at a ratio of 50:50:0.1 (v/v/v). The solution was stored at room temperature and used within 1 month.

- 1. **Preparation of Standard Stock Solutions**

All preparation was done under the yellow light.

Calibration samples and QC samples were prepared from separate stock solutions. All stock solutions were stored in amber glass vials in a refrigerator at 2°C to 8°C (actual temperature: 3.5°C to 6.6°C) and protected from light. The stock solutions were stored for a period of at maximum 83 days.

- - 1. **Calibration Stock Solution [A]**

ASP5354 chloride (approximately 11.1 mg*^1^, acceptable range: 10.0 mg to 12.2 mg, actual value: 11.02 mg and 10.69 mg) was accurately weighed and dissolved in water / acetonitrile (1:1, v/v) to obtain a nominal concentration of 2000 µg/mL.

*^1^: Target weight (mg)

= 10.0 (mg) ×1.112 (total correction factor)

Volume of water / acetonitrile (1:1, v/v) (mL)

= [actual weighed value (mg) / 11.1 (mg)] × 5 (mL)

- - 1. **QC Stock Solution [QA]**

ASP5354 chloride (approximately 11.1 mg*^1^, acceptable range: 10.0 mg to 12.2 mg, actual value: 10.67 mg and 10.08 mg) was accurately weighed and dissolved in water / acetonitrile (1:1, v/v) to obtain a nominal concentration of 2000 µg/mL.

*^1^: Target weight (mg)

= 10.0 (mg) ×1.112 (total correction factor)

Volume of water / acetonitrile (1:1, v/v) (mL)

= [actual weighed value (mg) / 11.1 (mg)] × 5 (mL)

- 1. **Preparation of Standard Working Solutions**

All preparation was done under the yellow light.

- - 1. **Calibration Working Solutions**

The calibration stock solution was serially diluted with water / acetonitrile (1:1, v/v) to prepare the following calibration working solutions as indicated in the table below.

The calibration working solutions of S5 to S11 were divided into 1 mL aliquots and stored. After use of the stored calibration working solution, the remaining solution was discarded and not be re-stored. These solutions were stored in amber glass vials in a refrigerator at 2°C to 8°C (actual temperature: 3.5°C to 6.6°C) and protected from light. The working solutions were stored for a period of at maximum 14 days.

The calibration working solutions of S1 to S4 were prepared at the time of use, and not stored.

| **Working Solution ID** | **Source Solution ID** | **Concentration of Source Solution (µg/mL)** | **Volume of Source Solution Aliquoted**  **(mL)** | **Final Volume of Working Solution**  **(mL)** | **Final Concentration of Working Solution**  **(µg/mL)** |
| --- | --- | --- | --- | --- | --- |
| S11 | A | 2000 | 0.1 | 10 | 20.0 |
| S10 | A | 2000 | 0.05 | 10 | 10.0 |
| S9 | A | 2000 | 0.045 | 10 | 9.00 |
| S8 | S11 | 20.0 | 1 | 10 | 2.00 |
| S7 | S10 | 10.0 | 1 | 10 | 1.00 |
| S6 | S10 | 10.0 | 0.5 | 10 | 0.500 |
| S5 | S8 | 2.00 | 1 | 10 | 0.200 |
| S4 | S7 | 1.00 | 0.5 | 5 | 0.100 |
| S3 | S6 | 0.500 | 0.5 | 5 | 0.0500 |
| S2 | S5 | 0.200 | 0.5 | 5 | 0.0200 |
| S1 | S4 | 0.100 | 0.5 | 5 | 0.0100 |

- - 1. **QC Working Solutions**

The QC stock solution was serially diluted with water / acetonitrile (1:1, v/v) to prepare the following QC working solutions as indicated in the table below.

These QC working solutions were divided into 1 mL aliquots and stored. After use of the stored QC working solution, the remaining solution was discarded and not be re-stored.

These solutions were stored in amber glass vials in a refrigerator at 2°C to 8°C (actual temperature: 3.5°C to 6.6°C) and protected from light. The working solutions were stored for a period of at maximum 14 days.

| **Working Solution ID** | **Source Solution ID** | **Concentrations of Source Solution (µg/mL)** | **Volume of Source Solution Aliquoted**  **(mL)** | **Final Volume of Working Solution**  **(mL)** | **Final Concentration of Working Solution**  **(µg/mL)** |
| --- | --- | --- | --- | --- | --- |
| Q3 | QA | 2000 | 0.2 | 5 | 80.0 |
| Q2 | Q3 | 80.0 | 0.5 | 10 | 4.00 |
| Q1 | Q2 | 4.00 | 0.75 | 10 | 0.300 |

- 1. **Preparation of IS Solutions**

All preparation was done under the yellow light.

- - 1. **IS Stock Solution [IA]**

AS3366010-CL (IS) (approximately 10.0 mg, acceptable range: 9.0 mg to 11.0 mg, actual value: 10.13 mg and 10.35 mg) was accurately weighed and dissolved in water / acetonitrile (1:1, v/v) to obtain a nominal concentration of 1000 µg/mL. The IS stock solutions were used immediately after preparation.

Volume of water / acetonitrile (1:1, v/v) (mL)

= [actual weighed value (mg) / 10.0 (mg)] × 10 (mL)

- - 1. **IS Working Solutions**

The IS stock solution was serially diluted with water / acetonitrile (1:1, v/v) to prepare the following IS working solutions as indicated in the table below.

When storing, the IS working solutions were divided into 1 mL aliquots and stored in amber glass vials. After use of the stored IS working solution, the remaining solution was discarded and not be re-stored. These solutions were stored in amber glass vials in a refrigerator at 2°C to 8°C (actual temperature: 3.5°C to 6.6°C) and protected from light. IS3 was stored for a period of at maximum 68 days. IS1 and IS2 were stored for a period of at maximum 28 days.

| **Working Solution ID** | **Source Solution ID** | **Concentrations of Source Solution (µg/mL)** | **Volume of Source Solution Aliquoted**  **(µL)** | **Final Volume of Working Solution**  **(mL)** | **Final Concentration of Working Solution**  **(µg/mL)** |
| --- | --- | --- | --- | --- | --- |
| IS3 | IA | 1000 | 1000 | 10 | 100 |
| IS2 | IS3 | 100 | 125 | 10 | 1.25 |
| IS1 | IS3 | 100 | 25 | 10 | 0.250 |

- 1. **Preparation of Calibration and QC Samples**

Calibration and QC sample were prepared in the same matrix and anticoagulant as expected for study samples.

- - 1. **Preparation of Calibration Samples**

Preparation of calibration standards was performed in ice water bath.

The calibration samples were freshly prepared on the day of use by adding 400 µL of water / formic acid (100:0.1, v/v) and 25 µL each of the individual calibration working solutions to each of 250 µL of pooled blank plasma to prepare 1.00, 2.00, 5.00, 10.0, 20.0, 50.0, 100, 200, 900, and 1000 ng/mL of ASP5354 in plasma.

| **Calibration Sample**  **ID** | **Source Solution ID** | **Concentration of Source Solution (µg/mL)** | **Volume of Source Solution Aliquoted**  **(µL)** | **Volume of Blank Plasma (µL)** | **Calibration Sample Concentration (ng/mL)** |
| --- | --- | --- | --- | --- | --- |
| STD1 | S1 | 0.0100 | 25 | 250 | 1.00 |
| STD2 | S2 | 0.0200 | 25 | 250 | 2.00 |
| STD3 | S3 | 0.0500 | 25 | 250 | 5.00 |
| STD4 | S4 | 0.100 | 25 | 250 | 10.0 |
| STD5 | S5 | 0.200 | 25 | 250 | 20.0 |
| STD6 | S6 | 0.500 | 25 | 250 | 50.0 |
| STD7 | S7 | 1.00 | 25 | 250 | 100 |
| STD8 | S8 | 2.00 | 25 | 250 | 200 |
| STD9 | S9 | 9.00 | 25 | 250 | 900 |
| STD10 | S10 | 10.0 | 25 | 250 | 1000 |

- - 1. **Preparation of QC Samples**

Preparation of QC samples was performed in ice water bath.

The QC samples were prepared as bulk solutions. Pooled blank plasma was put into separate PP tubes, followed by the addition of each QC working solution as indicated in the table below. The samples were mixed well. When the samples were stored, 300 µL portions of each QC samples were aliquoted into pre-labeled micro tubes. All QC samples were stored in a freezer at −70°C or below (actual temperature: −88.1°C to –70.1°C). The QC samples were stored for a period of at maximum 364 days.

| **QC**  **Sample ID** | **Source Solution ID** | **Concentration of Source Solution (µg/mL)** | **Volume of Source Solution**  **Aliquoted (µL)** | **Volume of Blank Plasma (µL)** | **QC Sample Concentration (ng/mL)** |
| --- | --- | --- | --- | --- | --- |
| LQC | Q1 | 0.300 | 18 | 1782 | 3.00 |
| MQC | Q2 | 4.00 | 18 | 1782 | 40.0 |
| HQC | Q3 | 80.0 | 18 | 1782 | 800 |

- 1. **Dilution of Samples**

When the assay value of a study sample was expected to exceed the highest concentration on the calibration curve, the sample was diluted 10 or 100-fold with pooled blank plasma as indicated in the table below and measured.

| **Dilution Factor** | **First dilution** | | **Second dilution** | |
| --- | --- | --- | --- | --- |
|  | **Volume of Study**  **Sample (µL)** | **Volume of Blank Matrix (µL)** | **Volume of First Dilution Aliquoted (µL)** | **Volume of Blank Matrix (µL)** |
| 10 | 25 | 225 |  |  |
| 100 | 25 | 225 | 25 | 225 |

- 1. **Sample Pre-treatment**
     1. **Sample Pre-treatment Procedure**

Samples included contamination and carryover samples (reagent blank, matrix blank [matrix blank spiked without IS], and control zero [matrix blank spiked with IS]) with a set of calibration and QC samples were treated in accordance with the following procedures:

- - - - 250 µL of each parent aliquot of the pooled blank plasma, QC samples and study samples were added to each micro tube in ice water bath. For reagent blank, 250 µL of water was added in ice water bath.
      - Water / formic acid (100:0.1, v/v) (400 µL) was added to each of the samples described above in ice water bath.
      - Calibration working solution (25 µL) was added to each calibration sample as described in Sec[tion 5.11.1](#_bookmark37) [Preparation of Calibration Samples](#_bookmark37) in ice water bath.
      - Water / acetonitrile (1:1, v/v) (50 µL) was added to the reagent blank and the matrix blank in ice water bath.
      - Water / acetonitrile (1:1, v/v) (25 µL) was added to the control zero, QC samples and study samples in ice water bath.
      - IS working solution [IS1 (0.250 µg/mL)] (25 µL) was added to each calibration sample, the control zero, QC samples and study samples in ice water bath.
      - All tubes were mixed with an Eppendorf MixMate (preset value: 1500 rpm, 1 minute) and centrifuged at 20000×*g* for 10 minutes set at 4°C.
      - Solid-phase extraction plate (OASIS HLB µElution plate) was preconditioned successively with methanol (200 µL) and water (200 µL).
      - Each supernatant was applied to each well of the OASIS HLB µElution plate.
      - After washing with water / methanol (9:1, v/v) (200 µL) by centrifugation, the analyte and the IS were eluted with methanol (25 µL × 2) by centrifugation into a 1 mL plate added water (50 µL) (preset value of centrifugation: 200×*g*, 2 minutes, 4°C).
      - Each mixture was shaken with an Eppendorf MixMate (preset value: 1000 rpm, 30 seconds).
      - Transfer into HPLC vial.
      - A 8-µL portion of the solution was injected into the LC-MS/MS system.
    1. **Flow Chart of Sample Pre-treatment Procedure**

The following flow chart shows the sample treatment procedure:

|  | | In ice water bath | | | | |
| --- | --- | --- | --- | --- | --- | --- |
|  | | Starting material | Water / formic acid (100:0.1,  v/v) (µL) | Calibration working solution (µL) | Water / acetonitrile (1:1, v/v) (µL) | IS working solution (IS1) (µL) |
| Blank | | Pooled blank plasma (250 µL) or water (250 µL) | 400 | — | 50 | — |
| Calibration samples | | Pooled blank plasma (250 µL) | 400 | 25 | — | 25 |
| Control zero | | Pooled blank plasma (250 µL) | 400 | — | 25 | 25 |
| QC samples | | QC sample (250 µL) | 400 | — | 25 | 25 |
| Study samples | | Study sample (250 µL) | 400 | — | 25 | 25 |
|  |  | | | | | |

1. Mix with an Eppendorf MixMate (preset value: 1500 rpm, 1 minute)
2. Centrifuge (preset value: 20000×*g*, 10 minutes, 4°C)
3. Load supernatant onto OASIS HLB µElution plate
   1. Preconditioned with methanol (200 µL) and water (200 µL)
4. Wash: water / methanol (9:1, v/v) (200 µL)
   1. (by centrifugation, preset value: 200×*g*, 2 minutes, 4°C)
5. Add water (50 µL) to a 1 mL plate
6. Elute to a 1 mL plate: methanol (25 µL×2)
   1. (by centrifugation, preset value: 200×*g*, 2 minutes, 4°C)
7. Shake with an Eppendorf MixMate (preset value: 1000 rpm, 30 seconds)
8. Transfer into HPLC vial
9. Inject into the LC-MS/MS system (8 µL)
   1. **HPLC Conditions**

HPLC system: NANOSPACE NASCA 2 system (OSAKA SODA)

Analytical column: CAPCELL PAK ADME

(2.1 mm I.D. × 20 mm, particle size 2 µm) (OSAKA SODA)

Mobile phase A: Water / formic acid (100:0.1, v/v)

Mobile phase B: Acetonitrile / formic acid (100:0.1, v/v)

| Gradient: | Time | Flow Rate | Mobile Phase | Mobile Phase |
| --- | --- | --- | --- | --- |
|  | (min) | (µL/min) | B (%) | A (%) |
|  | Init | 200 | 25 | 75 |
|  | 8.8 | 200 | 40 | 60 |
|  | 8.9 | 400 | 90 | 10 |
|  | 9.4 | 400 | 90 | 10 |
|  | 9.5 | 400 | 25 | 75 |
|  | 11.0 | 400 | 25 | 75 |

Wash solvent: Water / acetonitrile / formic acid (50:50:0.1, v/v/v)

Column temperature: 60°C (preset temperature)

Autosampler temperature: 5°C (preset temperature)

Injection volume: 8 µL

Run time: 11.0 min

Retention time for ASP5354: 3.9 min (3.3 – 4.5 min)

Retention time for IS: 3.9 min (3.3 – 4.5 min)

- 1. **MS/MS Conditions**

Mass spectrometer: QTRAP6500 (SCIEX)

Software: Analyst 1.6.2 (SCIEX)

Ionization: ESI (Turbo ion spray)

Ion polarity: Positive

Scan mode: MRM

Ion Spray voltage: 4500 V

Temperature (TEM): 400°C

Nebulizer gas (GS1): 50 psi

Turbo gas (GS2): 55 psi

Curtain gas (CUR) (nitrogen): 30 psi

Collision gas (CAD) (nitrogen): 12

Entrance potential (EP): 10 V

Valve position: Time (min) Valve Position

0.0 A (Divert)

2.0 B (MS)

6.0A (Divert)

Monitored ion, DP, CE, CXP and dwell time:

| Name | Precursor Ion | Product Ion | DP | CE | CXP | Dwell Time |
| --- | --- | --- | --- | --- | --- | --- |
|  | (*m/z*) | (*m/z*) | (V) | (V) | (V) | (msec) |
| ASP5354 | 1015 | 325 | 70 | 26 | 21 | 745 |
| IS | 1019 | 717 | 81 | 33 | 30 | 245 |

**Integration settings**

Integration algorithm: Intelliquan

- 1. **System Suitability**

System suitability was evaluated by injecting the standard solution at LLOQ level with the IS (n=6) to ensure the system was working properly prior to the start of an analytical run or a consecutive series of runs.

The retention times, peak shapes, and analyte and the IS background levels were evaluated for consistency.

The system was considered to be acceptable if the precision (CV) of the IS peak areas and the peak area ratios does not exceed 20.0%.

- - 1. **Preparation of System Suitability Check Sample**

The calibration working solution [S3 (0.0500 µg/mL); 5 µL], the IS working solution [IS2 (1.25 µg/mL); 5 µL], water (45 µL), and methanol (45 µL) were put into a micro tube to prepare a system suitability check sample for injection into LC-MS/MS system. A 8-µL portion of the solution was injected into LC-MS/MS system.

- 1. **Contamination and Carryover**

Samples to monitor contamination and carryover were incorporated into each analytical run.

Contamination during sample processing was investigated by injecting a reagent blank, a matrix blank and a control zero.

Carryover from the analytical equipment was investigated within each analytical run. The extent of the carryover was determined by injecting blank matrix samples (n=1) immediately after the ULOQ (the highest calibration) sample.

The LLOQ (the lowest calibration) sample in the same run was used as the reference. Contamination and carryover acceptance criteria are as follows:

1. The ratio of interference peaks versus the analyte peak at LLOQ has to be less than 0.200,
2. The ratio of interference peaks versus the IS peak has to be less than 0.0500.

**RESULTS**

**Supplemental Table S1. Participant Demographics and Baseline Characteristics**

|  |  | **ASP5354** | | | | |  |
| --- | --- | --- | --- | --- | --- | --- | --- |
|  | **Placebo (n=10)** | **0.1 mg**  **(n=4)** | **0.5 mg**  **(n=4)** | **2 mg**  **(n=4)** | **8 mg**  **(n=4)** | **24 mg**  **(n=4)** | **Overall (N=30)** |
| Age, mean (SD) years | 44 (11.2) | 43 (13.2) | 45 (15.2) | 44 (11.0) | 43 (10.7) | 41 (14.2) | 43 (11.3) |
| Sex, n (%) |  |  |  |  |  |  |  |
| Female | 5 (50.0) | 2 (50.0) | 2 (50.0) | 2 (50.0) | 2 (50.0) | 2 (50.0) | 15 (50.0) |
| Male | 5 (50.0) | 2 (50.0) | 2 (50.0) | 2 (50.0) | 2 (50.0) | 2 (50.0) | 15 (50.0) |
| Race |  |  |  |  |  |  |  |
| White | 7 (70.0) | 2 (50.0) | 3 (75.0) | 2 (50.0) | 2 (50.0) | 4 (100.0) | 20 (66.7) |
| Black or African American | 3 (30.0) | 2 (50.0) | 0 | 2 (50.0) | 1 (25.0) | 0 | 8 (26.7) |
| Other^a^ | 0 | 0 | 1 (25.0) | 0 | 1 (25.0) | 0 | 2 (6.6) |
| Hispanic or Latino, n (%) | 4 (40.0) | 0 | 2 (50.0) | 1 (25.0) | 1 (25.0) | 1 (25.0) | 9 (30.0) |
| Weight, mean (SD) kg | 76.7 (13.5) | 82.8 (18.0) | 75.5 (11.2) | 78.4 (15.6) | 78.5 (11.1) | 77.7 (16.4) | 78.0 (13.2) |
| BMI, mean (SD) kg/m^2^ | 26.6 (3.5) | 29.0 (2.9) | 28.0 (1.1) | 26.5 (4.2) | 26.9 (2.8) | 27.5 (2.2) | 27.3 (3.0) |

^a^Includes Asian and Native Hawaiian or Pacific Islander.

BMI, body mass index; SD, standard deviation.

**REFERENCE**

1. Fushiki H, Yoshikawa T, Matsuda T, Sato T, Suwa A. Preclinical development and validation of ASP5354: A near-infrared fluorescent agent for intraoperative ureter visualization. *Molecular Imaging and Biology.* 2021; May 11 online ahead of print.
