# Supplementary material for: Randomized, Double‐Blind, Controlled Study to Evaluate Safety and Pharmacokinetics of Single Ascending Doses of ASP5354, an Investigational Imaging Product, in Healthy Adult Volunteers
Source: Clin Pharmacol Drug Dev. 2021 Aug 23;10(12):1460–8. doi: 10.1002/cpdd.1013 (PMC9292347; doi:10.1002/cpdd.1013)
Supplement: Supplementary file 2 — Supporting Information [file CPDD-10-1460-s001.docx]

**Table S1. Participant Demographics and Baseline Characteristics**

|  |  | **ASP5354** | | | | |  |
| --- | --- | --- | --- | --- | --- | --- | --- |
|  | **Placebo (n=10)** | **0.1 mg**  **(n=4)** | **0.5 mg**  **(n=4)** | **2 mg**  **(n=4)** | **8 mg**  **(n=4)** | **24 mg**  **(n=4)** | **Overall (N=30)** |
| Age, mean (SD) years | 44 (11.2) | 43 (13.2) | 45 (15.2) | 44 (11.0) | 43 (10.7) | 41 (14.2) | 43 (11.3) |
| Sex, n (%) |  |  |  |  |  |  |  |
| Female | 5 (50.0) | 2 (50.0) | 2 (50.0) | 2 (50.0) | 2 (50.0) | 2 (50.0) | 15 (50.0) |
| Male | 5 (50.0) | 2 (50.0) | 2 (50.0) | 2 (50.0) | 2 (50.0) | 2 (50.0) | 15 (50.0) |
| Race |  |  |  |  |  |  |  |
| White | 7 (70.0) | 2 (50.0) | 3 (75.0) | 2 (50.0) | 2 (50.0) | 4 (100.0) | 20 (66.7) |
| Black or African American | 3 (30.0) | 2 (50.0) | 0 | 2 (50.0) | 1 (25.0) | 0 | 8 (26.7) |
| Other^a^ | 0 | 0 | 1 (25.0) | 0 | 1 (25.0) | 0 | 2 (6.6) |
| Hispanic or Latino, n (%) | 4 (40.0) | 0 | 2 (50.0) | 1 (25.0) | 1 (25.0) | 1 (25.0) | 9 (30.0) |
| Weight, mean (SD) kg | 76.7 (13.5) | 82.8 (18.0) | 75.5 (11.2) | 78.4 (15.6) | 78.5 (11.1) | 77.7 (16.4) | 78.0 (13.2) |
| BMI, mean (SD) kg/m^2^ | 26.6 (3.5) | 29.0 (2.9) | 28.0 (1.1) | 26.5 (4.2) | 26.9 (2.8) | 27.5 (2.2) | 27.3 (3.0) |

^a^Includes Asian and Native Hawaiian or Pacific Islander.

BMI, body mass index; SD, standard deviation.
